# Innate immune response to *Streptococcus suis* infection: advances and future challenges

**DOI:** 10.1186/s13567-026-01838-3

**Published:** 2026-10-11

**Authors:** François Meurens, Fanny Renois, Jianzhong Zhu, Cynthia Calzas, Jerry Mark Wells, Marcelo Gottschalk, Mariela Segura

**Affiliations:** 1 Research Group On Infectious Diseases in Production Animals (GREMIP) and Swine and Poultry Infectious Diseases Research Center, Faculty of Veterinary Medicine, University of Montreal, Saint-Hyacinthe, QC Canada; 2 Department of Veterinary Microbiology and Immunology, Western College of Veterinary Medicine, University of Saskatchewan, Saskatoon, S7N5E3 Canada; 3 College of Veterinary Medicine, Yangzhou University, Yangzhou, 225009 China; 4 Host-Microbe Interactomics, Animal Sciences, Wageningen University, 6708 WD Wageningen, The Netherlands

**Keywords:** *Streptococcus suis*, pig, immune response, zoonosis, phagocytes, cytokines, virulence factors

## Abstract

*Streptococcus suis* is a pathogenic bacterium primarily found in pigs, where it causes sudden death and meningitis, but it is also capable of causing septic shock and meningitis in humans, being the most important cause of adult bacterial meningitis in Southeast Asia. As the host’s innate immune system serves as the first line of defense against microbial invaders, it is crucial to understand the intricate relationship between the bacterium and this system. Despite an increasing number of studies performed in the last 20 years, the pathogenesis of *S. suis* infection is not fully understood, and there is still an absence of effective vaccines on the market. Recent population-genomic studies have further highlighted that pathogenic *S. suis* is not a single uniform entity but comprises multiple lineages with distinct genome content and disease associations, raising the possibility that innate immune evasion mechanisms may not be equivalent across lineage. In the current article, we conducted a comprehensive review of the literature, focusing on the innate response developed mainly by phagocytes against this Gram-positive bacterium. Our aim was to summarize the last findings and to identify the main gaps in knowledge to guide future research efforts toward areas that require further investigation.

## Introduction

*Streptococcus suis* (*S. suis*), a major porcine pathogen first reported by Jansen and Van Doorsen in the Netherlands [1], is responsible for important economic losses in the swine industry, as well as being a zoonotic agent, with deadly outbreaks reported in Southeast Asian countries [2–4]. This encapsulated extracellular bacterium is highly invasive. After penetration of host mucosal barriers, *S. suis* can reach and survive in the blood compartment, and finally invade multiple organs including spleen, liver, kidney, lung, and heart [4]. In the case where the host survives systemic infections, the bacteria are able to cross the brain microvascular endothelial cells (BMECs) at the blood–brain barrier (BBB) and/or the epithelial cells of the choroid plexus at the blood–cerebrospinal fluid barrier to gain access to the central nervous system (CNS). Septicemia, meningitis, endocarditis, and arthritis are the most common forms of *S. suis* disease [4]. The pathogenicity of *S. suis* is mediated by a large collection of proposed virulence factors/markers, including capsular polysaccharide (CPS), suilysin (SLY), extracellular factor (EF), muramidase-released protein (MRP), adhesins, and enzymes [5–12]. Yet, few of these factors have been fully characterized, and only the CPS has been suggested as a critical virulence factor. Among the 29 serotypes described, serotype 2 is the most frequently isolated from both diseased pigs and humans, and, consequently, research has mostly focused on this serotype [4]. However, recent population-genomic studies show that pathogenic *S. suis* is not a homogeneous entity. A large-scale analysis of 3070 isolates identified ten broadly distributed pathogenic lineages that together accounted for most disease-associated isolates and differed in serotype, sequence type (ST), genome content, and association with systemic versus respiratory disease [13]. Importantly, these lineages also share conserved pathogenicity-associated genomic islands with predicted roles in metabolism of complex polysaccharides, extracellular matrix interaction, and cell adhesion, suggesting that some determinants of pathogenicity may be common across lineages. Nevertheless, most mechanistic studies of virulence and innate immune evasion have focused on serotype 2, particularly ST1-related strains, and the relative contribution of individual factors may differ among lineages. Thus, despite the increasing number of studies, the pathogenesis of *S. suis* infection remains incompletely understood, and attempts to control the disease are still hampered by the lack of an effective and broadly protective vaccine.

In this narrative review, we describe the innate immune response mounted by the host against *S. suis* with particular emphasis on phagocytes and on the role of proposed *S. suis* virulence factors/markers to host–cell interactions and immune evasion mechanisms. Mouse models reproduce some of the clinical and inflammatory features observed in pigs [4, 14–16] and have therefore been legitimately used alongside cells from pig and human origins to explore the immunopathogenesis of *S. suis* infection.

## *Streptococcus suis* interaction with the mucosal barriers

In pigs, *S. suis* usually colonizes the upper respiratory tract, in particular, the pharyngeal and palatine tonsils, where its diversity is high [17–19], although the alimentary and genital tracts can also be colonized [4]. Naso- and oropharyngeal carriage of *S. suis* has been documented mainly in high-risk groups such as butchers, abattoir and meat-processing workers, and swine-facility staff. Reported prevalence varies considerably, ranging from about 5% in Germany [20] and Mexico [21] to as high as 20% in Italy (serotype 2, detected from tonsil cultures) [22]. These differences likely reflect the sampling site (tonsil, pharynx, or nasal cavity), the detection method (culture versus PCR), and the specific work environment. Humans can also acquire infection through the gastrointestinal route after consumption of fresh, raw, or undercooked contaminated pork products [23, 24]. In pigs, the contribution of the gastrointestinal route to *S. suis* pathogenesis remains unclear. Although experimental studies have demonstrated that oral exposure can occasionally lead to systemic infection, the efficiency of this route appears limited, and the specific conditions that would allow substantial survival and translocation of *S. suis* through the gastric barrier have yet to be clearly defined [25–27]. The mucosa of respiratory and gastrointestinal tracts, composed primarily of epithelial cells, mucus, antimicrobial peptides, and the underlying lymphoid tissue, is the first line of defense of the host against pathogens. Colonization of the swine upper respiratory tract by *S. suis* may lead to an asymptomatic carriage but is also considered the first step for the development of an invasive disease in pigs characterized by hematogenous and lymphatic dissemination. It is still unknown how *S. suis*, usually found in very low quantities in the tonsils of pigs, manages to enter the tonsils [28]. Most likely, this occurs in the crypts where the normally stratified epithelium becomes only one-cell thick [29]. Both inadequate host immune responses and environmental factors, including the presence of other pathogens, are likely to play a role [8, 30, 31].

### Interaction of *Streptococcus suis* with mucosal epithelial cells

Experimental aerosol infections in pigs have demonstrated the presence of *S. suis* in the crypt epithelium and in the subepithelial lymphoid tissue of the palatine tonsils, suggesting that bacteria are able to breach the mucosal epithelial barrier [28, 32]. A previous review published in 2016 summarized knowledge of the initial steps of *S. suis* pathogenesis [8]. Since then, researches have revealed new mechanisms and model systems that might be critical for *S. suis* colonization and/or invasion of human and porcine mucosal barriers including the intestinal epithelium [33, 34]. Nevertheless, in vitro experiments investigating the capacity of the bacteria to adhere to and/or invade human or porcine epithelial cells remain controversial. Whereas some studies have shown an adhesion by well-encapsulated *S. suis* [35, 36], others have demonstrated that CPS expression could decrease [7, 25, 37–42] or completely inhibit adhesion [43]. Similarly, many studies indicate that *S. suis* generally has low or absent invasive capacity toward mucosal epithelial cells [7, 36, 38, 39]. As with adhesion, the presence of CPS seems to decrease or inhibit the invasion of epithelial cells by *S. suis* [7, 25, 39, 41, 43, 44]. One explanation is that CPS hinders bacterial molecules contributing to adhesion and/or invasion, with effects that vary between serotypes [8, 25, 37]. Indeed, “capsule-switch” panels showed that changing CPS serotype alters adhesion to, but not invasion of, porcine tracheal epithelial cells; this work explicitly links CPS composition to host–cell interactions [7].

Several adhesins expressed by *S. suis* have been described to interact in vitro with epithelial cells and/or components of the extracellular matrix, which forms an important interface between the epithelium and the underlying mucosal tissue [6, 8, 45, 46]. For example, a study reported that the type II histidine triad protein HtpsC of highly virulent *S. suis* serotype 2 facilitates adhesion and invasion of human and porcine epithelial cells by binding E-cadherin [47], while also facilitating binding to fibronectin and laminin in vitro, supporting its role as an adhesin [48]. The autolysin (Atl) of *S. suis* serotype 2 promotes dysfunction of swine tracheal epithelium by interacting with vimentin and favoring bacterial translocation [49], corroborating previous findings on the role of Atl in adhesion to human epithelial cells [50]. Binding of this protein to fibronectin was also reported [50]. *Streptococcus suis* nuclease A (SsnA), ornithine carbamoyltransferase (OTC), surface-associated arginine peptidase [51], and MRP [46] have been shown to be involved in the adhesion and/or invasion of the bacteria to human laryngeal epithelial cells (Hep-2) or porcine kidney epithelial cells (PK-15) [52–54]. OTC has also been suggested to have a role in biofilm formation [54], as it is also the case of Atl [50]. However, the in vivo relevance of biofilms during *S. suis* infection remains to be experimentally established. The mechanisms by which OTC (a metabolic enzyme) and SsnA (a cell wall-anchored extracellular DNase) influence adhesion is not clear: whether directly binding to host cells, altering bacterial physiology to enhance adhesion, or affecting cell surface structures is an open question. The protein MRP as well as several other *S. suis* adhesins (such as GAPDH and enolase) were shown to be multifunctional proteins that can bind to one or more different host proteins including fibrinogen, laminin, fibronectin, collagen, factor H, and/or plasminogen [45, 46, 55]. It has been suggested that binding to some of these molecules can modulate the interactions between *S. suis* and epithelial cells, yet contradictory results are reported depending on assay conditions. Since most of the studies have mainly evaluated the interactions of host proteins with purified recombinant *S. suis* adhesins, their actual contribution to colonization and disease pathogenesis by intact bacteria remains to be established. For instance, a laminin-binding protein (named Lmb, lipoprotein 103, CDS 0330, or AdcA II) was recently shown to play no role in the laminin-binding activity of *S. suis*, even when clearly exposed at the bacterial surface [56]. Indeed, Lmb as well as AdcA, are both involved in zinc acquisition. While one study suggested that both proteins are involved in invasion of human Hep-2 laryngeal epithelial cells [57], a second report revealed that the presence of Lmb does not influence bacterial adhesion to and invasion of porcine respiratory epithelial cells [56].

SLY, a thiol-activated hemolysin that targets cholesterol in the membrane of eukaryotic cells, has been demonstrated to exhibit a significant role in the invasion of human epithelial cells by *S. suis* [9]. However, virulent *S. suis* strains that do not produce SLY are still able to adhere to epithelial cells and generate disease in pigs. Thus, SLY-positive *S. suis* strains may use cytotoxicity and barrier disruption/paracellular translocation as mechanisms to breach the respiratory epithelia, whereas SLY-negative strains must rely on SLY-independent mechanisms [38, 58]. *S. suis* was shown to compromise the integrity of swine tracheal epithelial barrier and translocate across an epithelial cell monolayer. The SLY produced by *S. suis* appears to play a key role in the ability of the bacterium to damage this epithelial barrier, by affecting two major tight junction proteins (occludin and zonula occludens-1) [59]. In a well-differentiated porcine respiratory air–liquid interface culture (ALI), SLY promotes adherence, loss of ciliated cells, apoptosis, and invasion; cytolytic activity is required in this model [60]. A key result of this study was the high efficiency of *S. suis*-induced apoptosis and invasion upon infection under ALI conditions. These properties have been reported to be less efficient when analyzed with immortalized cells. Nonetheless, a study using mutant strains of *S. suis* expressing SLY without cytolytic activity has indicated that SLY-mediated invasion of human laryngeal epithelial cells by the bacteria could also occur in the absence of pore formation [61]. Finally, it should be noted that the amount of membrane-bound SLY, the cholesterol content of the cells, as well as their resealing capacity all affect the susceptibility of the different respiratory epithelial cells regarding the effects of SLY [62].

Environmental factors, such as the sugar composition, also possibly influence the in vitro ability of *S. suis* to interact with swine epithelial cells by regulating genes coding described/putative adhesins and invasins [8, 63]. The effect of environmental signals on the regulation of CPS expression and the subsequent interaction with epithelial cells needs to be better explored [8, 43].

The presence of other microorganisms may also influence how *S. suis* interacts with mucosal epithelial cells. In this regard, *S. suis* exhibits a better adhesion and/or invasion of swine tracheal epithelial cells previously infected with swine influenza H1N1 virus, which indicates that a weakening of host mucosal response may allow bacteria to cross the epithelial barrier [30, 31, 64]. Indeed, binding of *S. suis* to virus-infected cells or directly to the virus [64] can both play a role in the invasion and systemic dissemination of the bacteria [41]. The interaction between the influenza virus and *S. suis* serotype 2 is sialic-acid-dependent, the capsular sialic acid of *S. suis* being recognized by the hemagglutinin of the virus. When *S. suis* is pretreated with neuraminidase, the bacteria number found on influenza-infected newborn porcine tracheal epithelial cells is strongly reduced [64]. Using the aforementioned ALI cultured primary porcine respiratory epithelial cells, it was proposed that coinfection with swine influenza virus may compensate for the lack of SLY in the adherence and invasion process of SLY-negative serotype 2 *S. suis* [65]. By contrast with *Bordetella bronchiseptica*, which promotes *S. suis* infection through ciliostasis, enhanced adherence and colonization, and SLY-dependent cytotoxicity [65], previous infection with *Mycoplasma hyopneumoniae* and/or *Mycoplasma hyorhinis* does not affect *S. suis* adhesion or invasion of pig tracheal epithelial cells [39]. Still, in situations of co-infection, Vötsch et al. demonstrated that *B. bronchiseptica* promotes adherence and colonization using porcine precision-cut lung slices (PCLS) [66]. Consequently, this increases the colonization and the cytotoxic effects of *S. suis*, likely due to the reduction of ciliary activity. The cytotoxicity induced by *S. suis* is strictly dependent on the presence of SLY [66]. More recently, an in vivo study in pigs has shown that, although *Bordetella bronchiseptica* facilitates the colonization of *S. suis* serotype 2, this does not result in a more severe disease compared with infection with *S. suis* alone [67]. Interestingly, the eukaryotic-like serine-threonine kinase protein (StkP) located inside extracellular vesicles released by *Streptococcus pneumoniae* induces the autophagic degradation of host occludin, contributing to the disruption of the alveolar epithelial barrier and bacterial dissemination [68]. Whether *S. suis* extracellular vesicles can similarly affect epithelial tight-junction integrity remains unknown. Although *S. suis*-derived extracellular vesicles have recently been reported to be internalized by epithelial cells and macrophages, and to modulate host responses, their direct effects on mucosal epithelial barrier function have not yet been established.

Studies described above mostly used respiratory epithelial cells in vitro. Administration of *S. suis* in pigs, via oral route—using gastric-acid-resistant capsule—or through a duodenal canula, has evidenced that bacteria manage to translocate from the gut to mesenteric lymph nodes and organs, suggesting the intestinal tract could be another infection route of the pathogen [25, 26, 63]. Finally, *S. suis* is also able to translocate across human intestinal epithelial cells in vitro mainly via a paracellular route, without disrupting monolayer integrity, confirming the zoonotic potential of *S. suis* via oral contamination [25, 34]. In these studies, expression of CPS impairs the adhesion and invasion of the bacteria [25, 34]. A study further demonstrated that the streptococcal adhesin protein 1 (SadP1) present in zoonotic *S. suis* isolates of clonal complex 1 contributes to binding to human intestinal epithelial cells and translocation across this cell barrier. It has been shown that SadP adhesin is recognized by Gb3/CD77 as its natural glycolipid host-cell receptor. However, SadP1 deletion does not entirely abolish bacterial adhesion, indicating that other bacterial surface-associated factors are likely involved in this process [69, 70]. Interestingly, it has been suggested that SadP variants might present different degrees of host specificity, notable for their adhesion to porcine intestinal epithelial cells [69]. More recently, using their human intestinal organoid model, Roodsant et al. demonstrated that the ability of *S. suis* to translocate across the enteroid barrier depends on the bacterial core genome composition and the presence of cells expressing Gb3 in the intestinal epithelium, SadP, and SLY being inessential for translocation [34].

### Role of host antimicrobial peptides and effector molecules in resistance to *S. suis* colonization and dissemination

Ciliated and goblet cells of epithelial surfaces, together with submucosal glands, are key players in innate mucosal immunity. Beyond providing a physical barrier, epithelial cells also form a potent biochemical shield by secreting mucus, antimicrobial molecules, and a variety of cytokines [71, 72]. Yet, the specific contribution of these effector molecules in limiting *S. suis* colonization and infection remains poorly understood.

Mucosal surfaces are protected by both secreted and membrane-associated mucins (MUC). In the intestine, goblet-cell-derived MUC2 is the dominant gel-forming mucin and contributes to a mucus layer that limits direct contact between bacteria and the epithelial surface [73, 74]. In the respiratory tract, secreted gel-forming mucins, mainly MUC5AC and MUC5B, form part of the airway mucus layer, which traps inhaled or aspirated microorganisms and is cleared by mucociliary transport [75, 76]. In both tissues, membrane-tethered mucins, including MUC1, MUC4, and MUC16, are heavily glycosylated components of the epithelial glycocalyx and may influence bacterial adhesion, signaling, and barrier protection. Many bacteria overcome these innate defense mechanisms by damaging cilia, altering mucin production or glycosylation, degrading mucins, or exploiting mucin glycans as adhesion targets or nutrient sources [74–76]. Although differentiated porcine respiratory ALI cultures and PCLS have been used to study *S. suis* interactions with the airway epithelium, including adhesion, invasion, apoptosis, ciliary effects, and coinfection, the direct interaction of *S. suis* with airway mucus, intestinal mucus, or specific secreted and membrane-bound mucins remains poorly defined. The best characterized example is ApuA, a cell-wall-anchored bifunctional amylopullulanase that promotes adhesion to porcine tracheal epithelial cells and binding to porcine gastric mucin in vitro [35, 77]. However, whether *S. suis* penetrates mucus, modifies mucin expression or glycosylation, degrades mucins, or exploits mucin glycans during colonization remains unknown.

Epithelial cells produce a variety of antimicrobial polypeptides, including the large enzymatic protein lysozyme and small cationic antimicrobial peptides (CAMPs) [71, 78]. While their general modes of action, such as direct microbicidal effects and chemotactic activity on leukocytes, are well characterized in both humans and swine, their specific role in limiting *S. suis* colonization and dissemination remains largely unexplored. 

The lysozyme, found in high concentrations in several bodily secretions including mucus [79], induces bacterial lysis by hydrolyzing the glycosidic bond between *N*-acetylglucosamine and *N*-acetylmuramic acid in peptidoglycan (PG) cell wall. To withstand lysozyme activity, many bacteria have developed strategies that modify PG residues near the enzyme’s cleavage site, thereby lowering its affinity for the substrate. Although such mechanisms have been identified in *S. suis* [80], a direct link between lysozyme sensitivity or resistance and the bacterium’s ability to colonize or disseminate has yet to be demonstrated. The fact that some of these bacterial features are not expressed during growth in normal laboratory culture medium [81] complicates our understanding of their role during colonization. Experimental infections in mice and pigs have demonstrated that an isogenic mutant strain of *S. suis* deficient in *pgdA* gene, which is involved in *N*-deacetylation of *N*-acetylglucosamine, exhibits an impaired virulence and systemic dissemination [81]. Comparative whole-genome analysis of a lysozyme-sensitive *S. suis* strain and its resistant derivatives identified *oatA*, which encodes a PG *O*-acetyltransferase, as an additional contributor to lysozyme resistance [80]. Interestingly, lysozyme resistance in *S. suis* shows considerable variability, with serotypes 1 and 2 generally more sensitive than serotype 9 isolates. Reduced susceptibility to the enzyme has been linked to diminished or absent CPS expression and autolysin activity [80]. Indeed, the proposed role of VraSR, an important two-component regulatory system, in *S. suis* resistance to lysozyme digestion, is likely linked to reduced CPS expression [82]. From these findings, it can be inferred that lysozyme-sensitive isolates may have a greater tendency to disseminate and cause systemic disease, whereas lysozyme-resistant strains might be better adapted for persistent mucosal colonization [83]. A comparable association between lysozyme resistance and successful colonization of mucosal surfaces has also been reported for *S. pneumoniae* [84].

CAMPs are ubiquitous and broadly effective components of host innate immunity [71, 78]. Electrostatic interactions of CAMPs with negatively charged phospholipids induce physical disruption of bacterial cell membranes resulting in pathogen killing. Defensins and cathelicidins are the two major families of CAMPs produced by mammal mucosal epithelial cells [71, 78]. In 2014, an in vitro experiment displayed the susceptibility of a Chinese *S. suis* serotype 2 strain to the porcine *β*-defensin 2 [85], known to be produced by intestinal epithelial cells [71, 86]. Several Gram-positive bacteria have developed mechanisms to prevent CAMPs from crossing bacterial cell wall by increasing the positive charge of lipoteichoic acids (LTA) by *D*-alanylation [87, 88]. In *S. suis*, the *dltA* gene is implicated in this chemical modification. The *ΔdltA* mutant strain of *S. suis* shows an attenuated virulence in pigs and mice, reflected by a diminution of bacteremia in blood and organs in infected animals [89]. Although the *ΔdltA* mutant strain exhibits a decreased resistance against bacterium- and frog-derived CAMPs in vitro, the sensibility toward those produced by porcine or human epithelial cells has not been assessed. For instance, only one in vitro study has examined the direct influence of CAMP sensibility of *S. suis* strains on the interaction with mucosal cells and suggested no difference of adhesion and invasion of porcine respiratory epithelial cells between wild-type (WT) and *ΔdltA* mutant strains [41]. Interestingly, the *ΔdltA* mutant strain presents lower adherence and invasion rates to porcine BMECs, which could explain the absence of clinical signs of meningitis in animals infected with this mutant. It is important to note that in addition to epithelial cells, neutrophils also produce lysozyme and CAMPs; the interaction between *S. suis* and these leukocytes will be described in the next sections. More recently, it has been shown that the CAMP LL37 is cleaved by the cysteine protease ApdS [90] and the extracellular endopeptidase O (PepO) of *S. suis* [91], impairing its bactericidal activity against *S. suis*. More broadly, cathelicidins can also modulate inflammatory recognition of bacteria: in whole-bacterium experiments with *Escherichia coli*, cathelicidin-2 killed bacteria while reducing Toll-like receptor (TLR) 2 and TLR4-dependent macrophage activation, and several mammalian cathelicidins inhibited responses to nonviable bacteria, illustrating that these peptides can combine antimicrobial and immunomodulatory functions [92]. In addition to direct antimicrobial effects, cathelicidins may also modulate inflammatory signaling during *S. suis* infection. For example, cathelicidin-2 pretreatment of murine macrophages reduced *S. suis*-induced cytokine responses and suppressed several signaling pathways, although the relevance of this mechanism to natural porcine mucosal colonization remains to be established [93].

Finally, mucosal epithelial cells promote recruitment and activation of immune cells, including neutrophils, monocytes, dendritic cells (DCs), and lymphocytes, via the secretion of cytokines and chemokines [72]. Whereas the ability of epithelial cells to produce those inflammatory mediators has been well demonstrated in response to several bacteria [72, 94], the inflammatory response of respiratory or intestinal epithelial cells induced by *S. suis* remains poorly investigated. Only a few studies have shown that *S. suis* induces IL-8/ CXCL8, IL-6, and TNF-*α* expression after in vitro incubation with porcine tracheal epithelial cells [39, 41, 95, 96]. Involvement of the nucleotide-binding oligomerization domain-containing protein (NOD) 2/NF-κB signaling pathway in cytokine production by these cells has been suggested [96]. The influence of cytokine and chemokine secretion by epithelial cells on colonization and/or dissemination of *S. suis* remains unknown. More recently, Weldearegay et al. [97] demonstrated, using PCLS, that lung tissue, including epithelial stromal and resident immune cell populations, responded in a manner comparable with cultured epithelial cells, with increased transcription of IL-1*β*, IL-6, IL-18, TNF, and CXCL8 [97]. In contrast, transcript levels of the tight junction proteins claudin-1 and occludin remained unchanged.

## *Streptococcus suis* interactions with professional phagocytes and complement resistance

Once infectious agents have crossed mucosal barriers, they face professional phagocytes and complement in deep tissues, lymphoid organs, and the bloodstream [98–100]. Neutrophils, monocytes, and macrophages constitute major cellular arms of the innate antibacterial defense and act together with complement and relevant antibodies to recognize, opsonize, internalize, and kill invading microorganisms. Under the influence of cytokines/chemokines produced in an inflammatory context by local cells, neutrophils become activated and migrate from blood capillaries into sites of infection. In a similar way, monocytes are recruited and can differentiate into macrophages or monocyte-derived DC-like cells, although many macrophage tissue populations are retained independently of monocyte recruitment. As of the myeloid lineage [98], macrophages, and neutrophils share several microbe-killing functionalities, including phagocytosis, the oxidative burst to generate oxygen radicles and antimicrobial factors. Antimicrobial molecules of neutrophil granules include proteins (lysozyme, lactoferrin, elastase, cathepsin G, myeloperoxidase, etc.) and CAMPs, which can act both within the phagolysosome and in the extracellular environment after degranulation [101, 102]. Neutrophils and macrophages also share immunomodulatory activities and cooperate in the orchestration of the innate and adaptive immune responses via the secretion of cytokines/chemokines [98, 99]. Both cells have overlapping expression of cell surface receptors for several chemokines and are thus often co-recruited under infectious/inflammatory situations. They also express receptors for immunoglobulin (Ig) and complement, which facilitate the recognition, internalization, and killing of opsonized pathogens with host serum components. However, as a consequence of lineage specialization and tissue adaptation, the two types of phagocytes have acquired distinctive but complementary features that confer different antimicrobial capacities, lifespans, and tissue distributions [98]. The antimicrobial capacity of neutrophils is higher compared with that of macrophages, and, while neutrophils are preferentially present in blood, macrophages are mostly tissue-resident cells [98–100, 103].

*S. suis* interacts with the complement system, monocytes, and granulocytes in the blood, and, even within a single serotype, significant variations in interactions are observed between strains, suggesting the existence of multiple pathotypes [104]. Efficient control of bacteremia is therefore critical to prevent systemic dissemination and septic shock. In the CNS, inflammatory monocytes contributed to exacerbation of *S. suis*-induced local inflammation, while neutrophils participate in bacterial burden control in a intracisternal murine model [105]. However, control may be limited in the blood when there is a high-level bacteremia [106, 107]. Nevertheless, depletion or genetic impairment of these cell populations did not prevent the development of clinical CNS disease, suggesting that resident CNS cells, rather than infiltrating monocytes or neutrophils alone, are major drivers of CNS inflammation and disease in this model [105].

### Interactions with neutrophils and role of pattern recognition receptors

Neutrophils are the predominant leukocytes in the bloodstream [98] and among the first cells recruited to sites of *S. suis* infection, such as respiratory mucosa [32]. Histopathological lesions of infected humans and/or pigs are also characterized by substantial neutrophilic infiltrate in the splenic red pulp and in the CNS [83, 108, 109]. A review has explored in detail the literature on *S. suis* and neutrophils, from their interaction at the cellular level to the roles and behavior of neutrophils in the infected host in vivo [107]. Several cellular factors are involved in neutrophil recruitment following *S. suis* infection, including the granulocyte colony-stimulating factor (G-CSF) that controls neutrophil recruitment in the blood [106, 107]. Vimentin, an intermediate filament protein, has been shown to influence neutrophil recruitment to the respiratory epithelium during *S. suis* serotype 2 infection in a mouse model [96]. In parallel, studies using a mouse model of *S. suis* serotype 2 meningitis revealed that the G-protein-coupled chemoattractant receptor Fpr2 promotes neutrophil influx into the CNS, while, in the context of streptococcal toxic shock-like syndrome, the same receptor contributes to reduced neutrophil recruitment [110].

In vitro experiments examining interactions between *S. suis* and neutrophils have mainly used serotype 2 and demonstrated that bacteria have developed several strategies to evade killing by interfering with both intra- and extracellular bactericidal activities of these cells [8, 107, 111]. Among several bacterial factors putatively involved in interactions with phagocytes, the CPS has been proven to be critical—nonencapsulated mutants are avirulent and rapidly eliminated from the bloodstream in both mouse and pig models of infection [5, 6]. Several reports on CPS-mediated resistance to in vitro killing by porcine or human neutrophils confirm this trait [107, 112, 113]. An increase in CPS thickness after in vivo bacterial growth is linked to a better resistance to killing by porcine neutrophils [114]. The precise mechanisms of *S. suis* CPS-mediated resistance to neutrophils are still poorly known and subject to technical limitations, including differences in viability and purity of neutrophils, careful discrimination between phagocytosis and killing, and are among other factors contributing to controversial results [107]. Nevertheless, the absence of CPS is correlated with a higher adherence to the surface of neutrophils [51] as well as internalization and/or killing [107]. It remains to be determined whether *S. suis* CPS hinders cell wall components essential to the recognition and the activation of phagocytic mechanisms by neutrophils. In addition, the capacity of CPS to actively inhibit the phagocytic signaling machinery of neutrophils, as observed with macrophages (see next section), needs to be explored. Finally, even though it has not been proven yet, CPS might also physically block access of antimicrobial molecules to the underlying bacterial cell membrane, as reported for *Neisseria meningitidis* CPS [115].

Overall, the role of complement-mediated opsonization in *S. suis* elimination by neutrophils (in the absence of specific antibodies) is a matter of debate and depends on technical variations, the origin of cells (human, mouse, swine), and the *S. suis* strains used, as discussed by Bleuzé et al. [107]. Nevertheless, in most studies, opsonization by complete naive serum has a limited effect on encapsulated *S. suis* killing and/or phagocytosis by neutrophils [107, 113, 116]. The exact contribution of CPS to complement resistance remains to be fully understood, and, more particularly, that of the sialic acid moiety in the case of sialylated serotypes (such as serotype 2). It has been shown in vitro that the CPS of *S. suis* serotype 2 is involved in the recruitment of factor H, a soluble complement regulatory protein; yet, this effect was independent of the presence of sialic acid [55]. This result is unexpected for a sialylated CPS [117]. Sialic acid of Group B *Streptoccocus* (GBS) CPS blocks activation of the alternative complement pathway, conferring resistance to opsonophagocytic killing [118, 119]. The lower concentration of sialic acid in *S. suis* CPS [120] or the difference in the chemical link between sialic acid and the adjacent sugar [117, 121] may explain the contrasting immunomodulatory effect of this sugar between the two streptococci. Nevertheless, C3b deposition on the bacterial surface is substantially enhanced in the absence of *S. suis* CPS, suggesting that the CPS is crucial for complement evasion [122, 123]. Interestingly, addition of the cytokines GM-CSF, TNF-*α*, and IL-8, known to promote phagocytic activity and expression of neutrophil receptors during bacterial infections [124–126], induces a significant increase in *S. suis* killing by swine neutrophils in vitro [113]. In contrast, a more recent study revealed that encapsulated *S. suis* impairs optimal porcine neutrophil functions that are not rescued by priming with colony-stimulating factors (G-CSF and GM-CSF) [127]. Therefore, host factors influencing encapsulated *S. suis* clearance by neutrophils deserve further studies.

In addition to CPS, SLY is another factor helping *S. suis* to escape from killing by neutrophils. First, SLY exhibits cytotoxicity toward porcine neutrophils and appears to be the only toxic factor produced by the bacteria toward these cells [113]. Furthermore, studies using chemical neutralization of SLY or a SLY-negative isogenic mutant (*Δsly*) strain support that this hemolysin allows bacteria to impede complement-dependent killing by porcine neutrophils [113] or reduces complement fixation on *S. suis* [123]. A similar action has been described for pneumolysin, a SLY-related toxin produced by *S. pneumoniae* [128]. Besides interfering with complement, SLY may also use other strategies to prevent neutrophil action. A direct interference with internalization is unlikely, as depicted by the resistance of well-encapsulated *Δsly* mutant strain [113]. However, SLY may inhibit migration of neutrophils to infection sites similarly to pneumolysin [129]. In this regard, a surface-associated subtilisin-like protease (SspA) induces degradation of IL-8 produced by BMECs, and it has been suggested to be a mechanism used by *S. suis* to invade CNS by affecting the recruitment of neutrophils to the site of inflammation [130]. Nevertheless, the role of SLY in facilitating *S. suis* escape of immune defenses seems to be less significant than that of the CPS. Indeed, it has been reported that multiplication of *S. suis* in murine blood ex vivo depends on CPS but not SLY expression [123]. Finally, SLY is reported to be a key virulence factor in mouse models, yet *Δsly* mutant strains are as virulent as the wild-type strain in porcine models of systemic or respiratory infections [131, 132].

Among other strategies developed by *S. suis* to evade complement, one example is the production of an IgM-degrading enzyme (Ide_*Ssuis*_), which specifically cleaves porcine IgM and limits activation of the classical complement pathway, consequently reducing killing by swine neutrophils [133–136]. A *S. suis* protein, named SntA, was shown to interact with C1q and directly activate classical and lectin pathways, resulting in complement consumption, a mechanism that may contribute to resistance to the bactericidal activity of human neutrophils [137]. More recently, the truncated hyaluronidase HylS’ fragment expressed by some virulent ST1 strains [138] was shown to reduce C3b deposition on the bacterial surface and partially impair *S. suis* killing by neutrophils. In addition, two different surface proteins of *S. suis*, Fhb and Fhbp, have so far been described for their capacity to bind factor H [55]. It should be noted that sequence and functional analyses revealed that Fhb actually corresponds to the aforementioned adhesin SadP [139]. Therefore, besides its Gb3-binding capacity, Fhb inhibits complement-mediated killing by human neutrophils by preventing complement deposition at the surface of the pathogen [140, 141]. However, in the presence of complement factors, a double mutant lacking both Fhb and Fhbp was similarly killed by pig blood when compared with the wild-type strain [55], suggesting that recruitment of factor H to the *S. suis* cell surface is multifactorial and redundant. Consequently, the role and contribution of Fhbp, Fhb, and other factor H-binding bacterial components in the interactions with neutrophils remain to be fully evaluated.

Other bacterial factors also contribute to *S. suis* immune evasion. For instance, *D*-alanylation of LTA reduces *S. suis* serotype 2 association with porcine granulocytes, their oxidative burst response, and complement deposition [87]. More recently, the *S. suis* VraSR two-component system was identified as a regulatory system contributing to the survival of *S. suis* in human blood through a resistance to neutrophil-mediated killing [82]. However, direct contribution remains difficult to separate from its effect on CPS expression, which was modulated by VraSR and might account for the observed phenotype. *S. suis* can produce a 5’nucleotidase (Ssads) able to convert adenosine monophosphate to adenosine, resulting in impaired human neutrophil bactericidal functions in vitro [142]. Another study suggested that Ssads might have an impact on mouse neutrophil functions in vivo using a transcriptomic analysis [143]. The bacterial surface protein HP0487 was shown to be involved in the adhesion to mouse, human, and porcine neutrophils and promotes resistance to phagocytosis by mouse neutrophils [144]. On the other hand, the secreted *S. suis* transglutaminase SsTGase was reported to enhance resistance to killing by human neutrophils [145]. The exact mechanisms underlying the effect of these numerous bacterial factors in modulating interactions with neutrophils remain to be fully elucidated.

Finally, several studies have shown that *S. suis* uses various strategies to resist against CAMPs produced by neutrophils. In vitro experiments have demonstrated that the aforementioned modifications of the cell wall components (PG *N*-deacetylation and LTA *D*-alanylation) may be strategies used by *S. suis* to evade antimicrobial molecules of neutrophils [81, 87, 89]. Both *ΔpgdA* and *ΔdltA* mutant strains present increased in vitro sensibility to killing by porcine neutrophils [81, 89]. In addition, the *pgdA* gene is upregulated upon in vitro interaction with these cells [81]. Whether cell wall modifications allow *S. suis* to resist intra- or extracellular killing by antimicrobial molecules remains unknown. In addition, a work investigating the activity of porcine neutrophilic secretions, containing lactoferrin and cathelicidin, against different porcine bacterial pathogens indicates a significantly lower bactericidal action against *S. suis* in comparison with *Escherichia coli* or *Actinobacillus suis* [102]. Thus, *S. suis* may preferentially impede extracellular activities of antimicrobial molecules; for example, in the context of neutrophil extracellular traps (NET) formation. NETs are extracellular fibers of DNA with embedded antimicrobial molecules and proteases released by activated neutrophils that trap and kill microbes [146]. Numerous studies reported neutrophil release of NETs in the presence of *S. suis* [127, 147] using mouse, swine, and human cells. From a mechanistic perspective, the *S. suis*-induced release of NETs by murine neutrophils required NADPH-oxidase dependent reactive oxygen species (ROS), myeloperoxidase, and activation of P38 mitogen-activated protein kinase (MAPK), extracellular signal-regulated kinase (ERK) 1/2, and protein kinase C (PKC) pathways [148]. Consistent with this ROS-dependent pathway, the *sodA*-encoded Mn-superoxide dismutase in neutrophil immune evasion: deletion of *sodA* increased ROS accumulation, mitochondrial disruption, GSDMD-N expression, and NET formation, and reduced *S. suis* serotype 2 survival in mouse neutrophil assays [149]. A heterogeneity exists among *S. suis* strains regarding NET production and their capacity to resist NET killing, including technical discrepancies and lack of appropriate controls, as discussed by Bleuzé et al. [127, 147]. Even if *S. suis* can be entrapped by NETs formed by activated neutrophils, DNA-nucleases SsnA and EndAsuis are able to disrupt the structure and/or protect bacteria against the extracellular antimicrobial activity of those NETs in vitro [150, 151]. In accordance with the role of ApdS in evasion from cathelicidin LL-37 killing, this protease impaired the bactericidal effects of human neutrophils and NETs [90]. However, the role of CPS in NET evasion is subject to controversy and ranges from a protective to an active role in NET induction [127, 147]. Finally, the ability of *ΔpgdA* and *ΔdltA* mutant strains to resist NETs-mediated antimicrobial activity warrants examination. In vivo, NETs are detected in the CSF and brain tissue of pigs infected by *S. suis*, and their formation or persistence undergoes a complex regulatory process, including host DNAse and cathelicidin PR-39 [152]. PR-39 may protect the NETs against bacterial nucleases [152].

Receptors participating in *S. suis* interaction with neutrophils have been poorly characterized, but pattern recognition receptors (PRRs) such as TLRs, lectins, and complement receptor (CR) are potential candidates [153, 154]. The involvement of TLR4 in the recognition of *S. suis* serotype 2, leading to the induction of NETs, was reported [148]. The triggering receptor expressed on myeloid cell 1 (TREM-1) is induced on macrophages and neutrophils [155] and amplifies TLR-initiated responses against pathogens. It has been suggested that TREM-1 signaling promotes host defense during the early stage of mouse infection with highly pathogenic *S. suis* serotype 2 [156], by partially affecting the neutrophil response. Additional research is required to clarify the role of TLR and TREM-1 pathways in neutrophil activation by *S. suis*.

The lectin site of complement receptor 3 (CR3) [157] and sialic acid-binding immunoglobulin-like lectin (Siglec)-9 expressed by human neutrophils are engaged in GBS CPS recognition and may thus also be receptors for *S. suis* CPS, as both molecules share very similar structure and composition [117]. Interestingly, recognition of sialic acid of GBS CPS by Siglec-9 dampens neutrophil killing functions, such as degranulation, oxidative burst, and NET formation, and promotes up regulation of the expression of the inhibitory cytokine IL-10 [158]. Studies on interactions between sialic acid-expressing *S. suis* CPS and neutrophil Siglecs would be pertinent. Still, regarding the formation of NETs, a recent report showed that the expression of ribosomal protein SA on the surface of *S. suis*-infected neutrophils enhanced their ability to secrete NETs [159], and, even more recently, it was shown that *S. suis* CPS participates in the release of NETs [127].

### Interactions with monocytes/macrophages and the role of pattern recognition receptors

Concomitantly to the efflux of neutrophils, the exposure of pigs and/or mice to *S. suis* is followed by the recruitment of macrophages in infection sites such as tonsillar mucosa-associated lymphoid tissue [32, 160] and meningeal lesions [15, 83]. This macrophage enrollment plays an ambiguous role in *S. suis* infection, since it may protect the host by preventing the entry of the pathogen in blood and brain, but it can also be diverted by the bacteria to allow dissemination in the infected organism. In addition, *S. suis* infection is associated with an uncontrolled production of inflammatory mediators, and macrophages are mainly present in the inflammatory exudates.

In vitro interaction assays between *S. suis* serotype 2 and mouse macrophages indicate that bacteria remain mainly extracellularly bound to the plasma membrane and protrusions of the cell surface [161]. Sialic acid of the CPS may be partially engaged in this adherence, yet other bacterial molecules might be also involved [161]. Considering that receptors implicated in *S. suis* binding to macrophages have not been well identified, engagement of Siglec receptors is worth investigation for those serotypes harboring sialic acid. The presence of SLY is correlated with high cytotoxicity toward macrophages, and SLY-positive strains may thus use macrophage destruction for evasion of host immune clearance and dissemination [161]. The presence of serum increased bacterial adhesion to macrophages. Complement would be partially implicated in the serum-enhanced binding of *S. suis* to these cells, yet this feature remains to be fully evaluated. LTA could also contribute to the interactions between *S. suis* and monocytes; the association of the bacteria with porcine monocytes being reduced by *D*-alanylation of its LTA in the absence of specific antibodies [87]. However, a recent study with serotype 2 showed that even though LTA is involved in bacterial division and general fitness, it would have a limited role in pathogenesis [162].

The low internalization rate of *S. suis* serotype 2 by mouse, human, and/or porcine monocytes and macrophages is mainly explained by the antiphagocytic properties of CPS [7, 163–167]. Remarkably, *S. suis* CPS does not act simply as an inert physical barrier against macrophages but is an active modulator of the phagocytic signaling machinery of these cells. Experiments using latex beads covalently linked with purified *S. suis* serotype 2 CPS or nonencapsulated mutant strains have demonstrated that CPS adherence to mouse macrophages induces the destabilization of lipid microdomains at the cell surface preventing lactosylceramide (LacCer) accumulation at the phagocytic cup known to act as a PRR [168]. This results in a blockade of downstream intracellular signaling pathways involved in phagocytosis such as inhibition of kinase activation, including phosphatidylinositol 3-kinase (PI-3K), Akt, and PKC*α*, by recruiting, at least in part, SH2 domain-containing tyrosine phosphatases [169]. It would be relevant to evaluate if the recruitment of others PRRs at the LacCer signaling platform, such as CR3 or CD11b/CD18, known to be implicated in the phagocytosis of nonopsonized particles [170], is also inhibited by *S. suis* CPS. A recently described CPS-independent mechanism further supports the idea that *S. suis* can actively manipulate macrophage phagocytosis. Yang et al. identified an Ihk/Irr two-component regulatory system that controls *pepO* expression; *pepO* then activates TLR2-dependent p38 and ERK1/2 signaling, inhibits actin polymerization, and thereby reduces macrophage phagocytosis and enhances bacterial virulence [171]. Finally, it is important to note that internalized *S. suis* is generally killed efficiently or survive only poorly in macrophages under the conditions tested [46, 166]. Thus, under nonopsonizing conditions, *S. suis* likely remains preferentially extracellular throughout infection, either free in circulation or associated but not ingested, to the surface of macrophages as stipulated by the modified Trojan horse theory [172].

**Figure 1:**
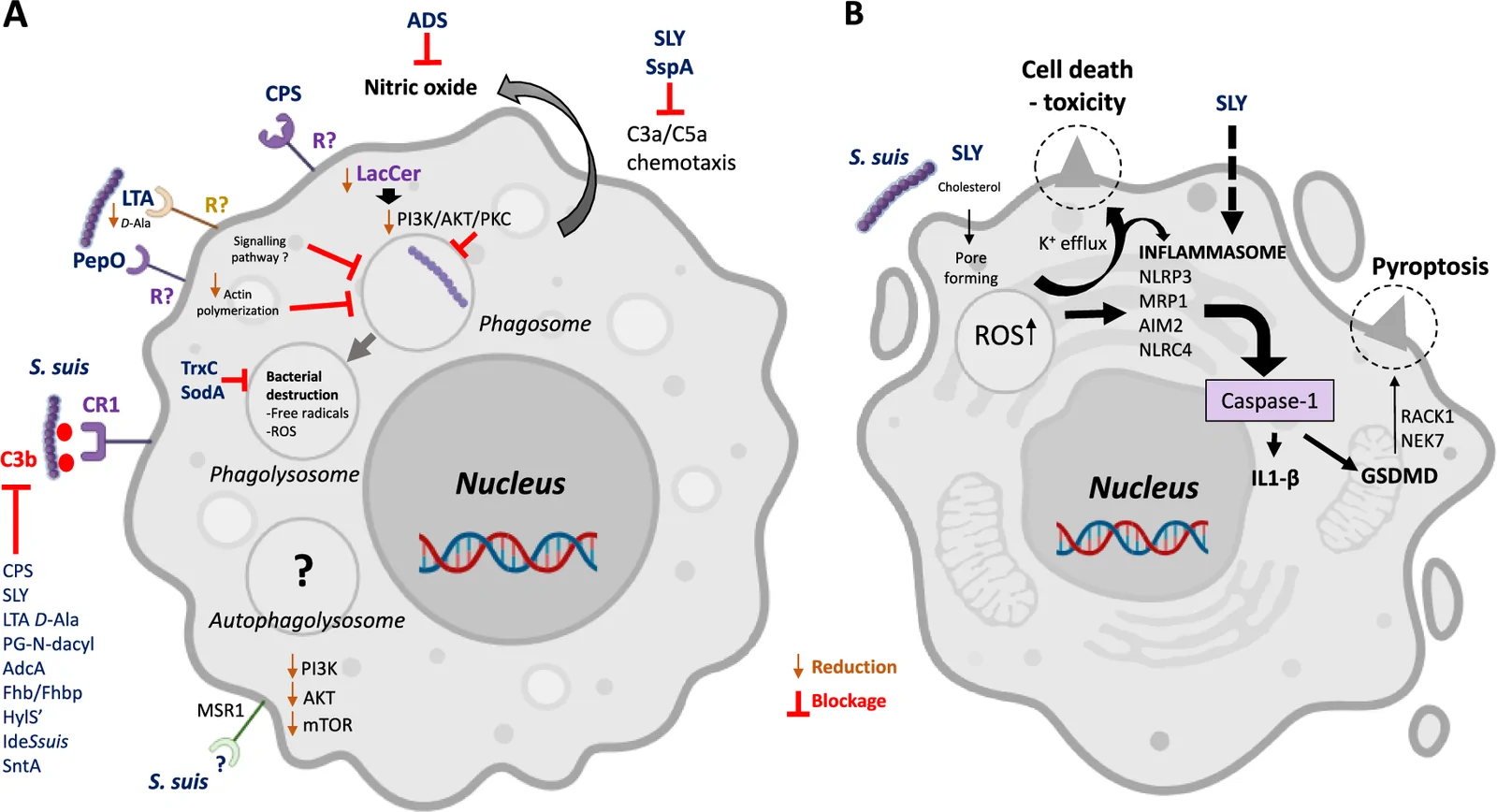
***Streptococcus suis*–host cell interactions**. **(A)** Adhesion, phagocytosis, and complement activation. **(B)** Cytotoxicity and pyroptosis. Schematic representation of identified interactions with target cells (either macrophage and/or dendritic cell). *AKT* protein kinase B, *AdcA* zinc-binding lipoprotein (named also as Lmb, lipoprotein 103, or CDS 0330), *ADS* arginine deiminase system, *AIM2* absent in melanoma 2 receptor, *C3a* complement component 3a, *C3b* complement component 3b, *C5a* complement component 5a, *CR1* complement receptor 1, *CPS* capsular polysaccharide, *Fhb/Fhbp* factor H-binding proteins, *HylS’* truncated hyaluronidase fragment, *IdeSsuis* Immunoglobulin M-degrading enzyme of *S. suis*, *IL-1β* interleukin 1 beta, *LacCer* lactosylceramide, *LTA* lipoteichoic acid, *LTA D-Ala* lipoteichoic acid *D*-alanylation, *mTOR* mechanistic target of rapamycin, *MRP* muramidase-released protein, *MSR1* macrophage scavenger receptor 1, *NEK7* NIMA-related kinase 7, *NLRC4* NLR family CARD domain containing 4, *NLRP3* NLR family pyrin domain containing 3, *PepO* endopeptidase O, *PG-N-dacyl* peptidoglycan *N*-deacetylation, *PKC* protein kinase C, *RACK1* receptor for activated C kinase 1, *ROS* reactive oxygen species, *SntA* surface-exposed protein, *SLY* suilysin, *SodA* superoxide dismutase A, *SspA* subtilisin-like protease, *TrxC* thioredoxin C. Created partially using BioRender.

Research has indicated that several virulence factors contribute to resistance to phagocytosis and/or killing by macrophages mediated by the complement system (Figure 1). However, experimental conditions lacking appropriate controls (such as presence/absence of serum and well-preserved sources of complement) can sometimes preclude a clear understanding of their actual contribution to the pathogenesis of infection. The zinc transporter AdcA was shown to interact with factor H and to inhibit C3b deposition on the bacterial surface, thereby contributing to resistance to opsonophagocytosis by murine macrophages in vitro [173]. It remains unclear whether a similar antiphagocytic effect would be observed in the absence of serum. Likewise, it is unknown whether the other *S. suis* zinc transporter, Lmb, plays a role in evasion of complement-mediated clearance, especially considering the absence of evidence for an antiphagocytic effect of this protein under nonopsonic conditions [56]. In the presence of human serum, neither Fhb nor Fhbp played a role in the resistance capacity of *S. suis* to complement-mediated opsonophagocytosis by human macrophages [55], while an effect was observed for the CPS. The truncated hyaluronidase fragment HylS’ [138] and the surface-exposed protein SntA [174] both reduce C3 deposition at the bacterial surface; however, their capacity to impair *S. suis* phagocytosis by murine macrophages was only evaluated in the absence of serum. Finally, SLY [174] and the subtilisin-like proteases SspA-1 and SspA-2 [175] contribute to the overall mechanisms of complement resistance by inhibiting monocyte chemotaxis mediated by C3a and C5a, albeit without proteolytic activity against C3a and C5a.

Most studies have addressed the antiphagocytosis mechanisms deployed by *S. suis*, and little information is available on *S. suis* capacity to resist the bactericidal effects of macrophages (Figure 1). Differentiated blood-derived monocytes of swine origin showed lower levels of killing than those observed with neutrophils [113]. The CPS was the main driver of resistance, independently of the presence of complete serum, whereas SLY was involved in bacterial resistance to complement-mediated killing by macrophages [113]. Additionally, it has been demonstrated that *S. suis* utilizes its superoxide dismutase, *sodA*, to neutralize the antimicrobial effects of ROS and reduce the autophagic response [176]. Still, to reduce autophagic activity in murine macrophages and promote *S. suis* survival, the thioredoxin C (TrxC) protein has demonstrated its ability to scavenge large amounts of free radicals, inhibit the PI3K-Akt-mTOR signaling pathway, and reduce the expression of MSR1, which is involved in autophagy [177] (Figure 1). *S. suis*-infected mouse macrophages produce nitric oxide (NO) [165, 168], known to participate in bactericidal functions [178]. Recently, it was suggested in a mouse model, that the streptococcal arginine deiminase system (ADS) is part of the strategies employed by *S. suis* serotype 2 to resist the bactericidal activity of macrophages by modulating the NO content of macrophages [179]. Several regulatory and enzymatic pathways as well as factors involved in the secretion and maturation of extracellular proteins (such as PrsA and Pdh) [180, 181] have been identified and suggested to be, at least indirectly, involved in the interactions between *S. suis* and the innate immune response, including macrophages. Since these factors modulate a panoply of putative bacterial virulence determinants, growth, fitness, and/or CPS expression, their actual role remains unclear.

As described above, *S. suis* disease is characterized by an exacerbated inflammatory response, which may be responsible for the clinical septic shock associated with septicemia as well as for the increase in the permeability of BBB with subsequent development of meningitis [4, 172]. However, at which stages of *S. suis* infection occurs the initiation of production of these mediators is still largely unidentified. *S. suis* serotype 2 induces significant release of proinflammatory and pyroptosis cytokines (TNF-*α*, IL-1*β*, IL-6), granulocyte-colony stimulating factor (G-CSF), and chemokines (IL-8/CXCL-8, MCP-1/CCL2, IP-10/CXCL10, MIP-2/CXCL2, CCL5) by mouse, human, and pig monocytes and macrophages—including alveolar macrophages—in a dose- and time-dependent fashion [165, 182–187]. This cytokine profile is compatible with a report indicating that *S. suis* infection induces an M1 macrophage polarization in infected mouse spleens [188].

**Figure 2:**
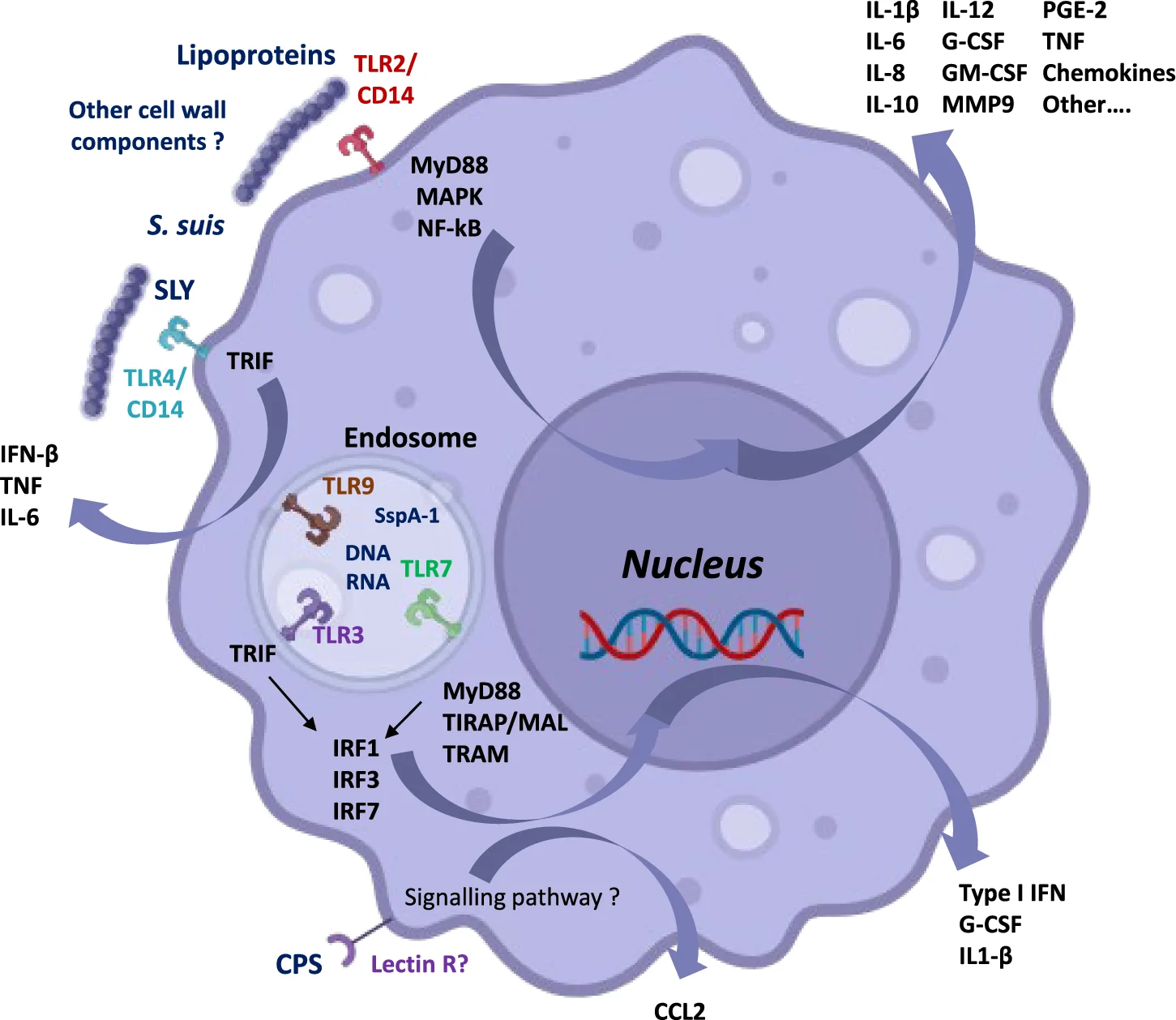
**Cytokine activation induced by *Streptococcus suis***. Schematic of pattern recognition receptor-mediated signaling pathways and cytokine production in response to *S. suis* infection. Identified interactions with target cells either macrophage and/or dendritic cell are shown. *CCL2* chemokine (C–C motif) ligand 2, *CD14* cluster of differentiation 14, *CPS* capsular polysaccharide, *G-CSF* granulocyte colony-stimulating factor, *GM-CSF* granulocyte–macrophage colony-stimulating factor, *IFN-β* interferon beta, *IL-1β* interleukin 1 beta, *IL-6* interleukin 6, *IRF* interferon regulatory factor, *MMP9* matrix metalloproteinase 9, *MyD88* myeloid differentiation primary response 88, *PGE2* prostaglandin E2, *SLY* suilysin, *SspA-1* subtilisin-like protease A-1, *TIRAP/MAL* TIR domain-containing adapter protein (MAL), *TLR* Toll-like receptor, *TRAM* TRIF-related adapter molecule, *TRIF* TIR-domain-containing adapter-inducing interferon-*β*, *TNF-α* tumor necrosis factor alpha, *type I IFN* type I interferon. Partially created using BioRender.

Studies of the interaction between purified *S. suis* components and human or mouse monocytes/macrophages have established that cell wall components of *S. suis* are mainly involved in cytokine and chemokine production [183, 187]. This is in agreement with the global higher secretion of these molecules by mouse and/or human monocytes and macrophages incubated with nonencapsulated mutant strains [165, 183]. Interestingly, hemoglobin, which may be released by the action of *S. suis* SLY on red blood cells, acts in synergy with *S. suis* cell wall to increase the inflammatory response of human macrophages [189]. LTA was initially considered a major inducer of cytokine production. However, subsequent studies demonstrated that lipoproteins, which frequently contaminate LTA preparations, are primarily responsible for the inflammatory responses observed in such experiments [162, 182]. Purified serotype 2 CPS solely stimulates the secretion of MCP-1/CCL2, which explains the diminished production of this chemokine by monocytes and macrophages incubated with the nonencapsulated mutant strains [183, 190]. Other factors can also contribute to macrophage inflammation such as the surface-associated arginine peptidase [51], SspA [191, 192], and SLY, the mechanisms of which are further described below (Figure 2).

As mentioned above, *S. suis* is generally considered an extracellular bacterium, and, even though small amounts of intracellular bacteria or bacterial-derived effectors molecules may stimulate the host inflammatory response intracellularly, this pathway is likely a weak contributor to cytokine secretion. Studies have reported that bacterial internalization is not required for the ability of *S. suis* to induce cytokine release by macrophages [182, 187]. Indeed, cytokine production by human monocytes and mouse macrophages cultured with whole *S. suis* and purified cell wall components is strongly dependent on TLR pathway via myeloid differentiation primary response 88 (MyD88), with an involvement of TLR2 [182, 183, 193, 194] (Figure 2). In vitro, the partial recognition of *S. suis* by TLR2 leads to the production of various cytokines including G-CSF, TNF-*α*, IL-1*β*, CCL2, IL-6, and IL-8 and is influenced by the CPS, cell wall components, and/or secretion effectors [37, 122, 182, 183, 192–198]. Lipoproteins are mainly implicated in TLR2-dependent recognition by macrophages in vivo [182]. In addition, CD14 also participates in cytokine secretion by *S. suis*-stimulated monocytes, and the region that interacts with the bacteria could be similar to the LPS-binding site [186]. In a study investigating the potential role of TLR4 in activation of murine and human macrophages, it has been reported that SLY induces TNF production following its recognition by this TLR [199]. In contrast, TLR4 was not involved in *S. suis*-induced G-CSF production by macrophages [182]. However, chemokine production is less stringently dependent on TLR2 and CD14 expression and may result from the engagement of multiple receptor pathways. MCP-1/CCL2 secretion is part of a proinflammatory cascade where TNF-*α* and IL-1*β* cytokines are produced early and modulate secretion of downstream chemokines [186]. The incomplete inhibition of this chemokine production by MyD88 mouse macrophages incubated with whole *S. suis* suggests that a third pathway, which is TLR independent, is involved in chemokine production. This is confirmed by similar MCP-1/CCL2 secretion levels by WT or MyD88 mouse macrophages stimulated with purified CPS. Both MAPK and NF-κB signal transduction pathways are involved in the proinflammatory response observed after *S. suis* or its purified components’ interactions with porcine, mouse, and/or human macrophages (Figure 2). Nevertheless, the role of MAPK kinases ERK, c-Jun N-terminal kinase (JNK), and p38 varies depending on the cytokine and/or the cell type [165, 182, 189, 199, 200].

Besides MyD88, an involvement of TRIF, an adaptor protein downstream of other TLRs, including TLR3, TLR4, and TLR5, was also observed in macrophages [182]. Since TLR4 is not generally involved in *S. suis* activation of cytokines, the role of other endosomal members of the TLR family was suggested. Indeed, dynamin-independent internalization of bacteria (or bacterial debris), followed by phagosome acidification, and *S. suis* DNA are part of the in–out signals leading to G-CSF production by macrophages [182]. Interestingly, transcription of TLR3 mRNA in brain myeloid cells of *S. suis* infected mice has been also described [15].

Type 1 interferon (IFN) signaling might also amplify TLR-initiated response against pathogens and enhance the secretion of chemokines and cytokines. However, the role of type I IFNs during *S. suis* infection appears to depend on both the virulence of the serotype 2 strain and the host cell type involved. One study reported low levels of IFN-*β* expression in mouse macrophages infected with *S. suis*, independently of the virulence of the strain [201]. Another study investigated the role of SspA-1, a type IV secretion effector produced by a highly virulent *S. suis* serotype 2 strain [192]. The authors showed that the endosomal TLR2 pathway mediates the type I IFN signaling induced by SspA-1 and the subsequent inflammatory response in human differentiated monocytes [192]. This response involved the adaptor proteins Mal, TRAM, and MyD88, together with downstream activation of IRF1 and IRF7. These findings are consistent with a previous report describing a nonproteolytic mechanism of human macrophage activation mediated by SspA [191]. However, since purified proteins were used in these studies, it remains to be determined whether a similar mechanism occurs during infection with whole bacteria.

Finally, research has explored the involvement of the inflammasome pathway in *S. suis* activation of macrophages and consequent streptococcal toxic shock-like syndrome, showing a role of NLRP3 complex over other inflammasomes [202, 203]. It is known that activation of the NLRP3 inflammasome leads to the secretion of IL-1*β*, which is mediated by caspase−1-cleaved gasdermin D (GSDMD), which in turn regulates a cell death mode termed pyroptosis. Pyroptosis is a form of programmed inflammatory cell death that can be triggered by pathogens and plays a critical role in the clearance of microbial infection [204–206]. Recently, a key role for RACK1 and NEK7 has been identified in *S. suis* serotype 2-induced pyroptosis in mouse macrophages [207]. The study also evidenced activation of the NLRP3 inflammasome and caspase−1, leading to the formation of mature GSDMD in *S. suis* serotype 2-infected mouse macrophages [207]. Further studies revealed that SLY appears to be one of the bacterial factors involved in the hyperactivation of the NLRP3 inflammasome, leading to capsapse−1 mediated IL-1*β* release and cytotoxicity in mouse and human macrophages [202, 203, 208]. It has been suggested that the K+ efflux and ROS generation might be involved in the SLY-induced NLRP3 inflammasome activation, implying a major role of the pore-forming activity of SLY in this pathway [202, 203, 208]. It should be noted that SLY also induces TNF-*α* and IL-6 secretion by human, mouse, and porcine monocytes and/or macrophages independently of its hemolytic activity [132, 161, 199] (Figure 2).

Besides cytokine and chemokine production, *S. suis* induces the release of other inflammatory mediators by macrophages, which are susceptible to participate in the pathophysiology of *S. suis*-induced disease. CPS and cell wall components of *S. suis* synergistically induce the release of prostaglandin E2 (PGE2) and matrix metalloproteinase 9 by human macrophages [209]. The production of these mediators was enhanced in a human brain microvascular endothelial cell/macrophage coculture model [210]. Interestingly, in addition to causing BBB permeability, PGE2 also interferes with the maturation and survival of B and T lymphocytes, and could potentially be another subterfuge of *S. suis* to escape from the host immunity. Once *S. suis* gains access to the CNS, the bacteria interact with meningeal and perivascular macrophages and microglia, the macrophage-like population within the brain [15, 98, 165]. Two studies have demonstrated that *S. suis* induces the release of the cytokines TNF-*α*, MCP-1/CCL2, IP-10/CXCL10, IL-1*β*, and IL-6, microbicidal NO, and enhances TLR2 and NOD2 expression by murine microglial cells in vitro [165, 168]. In addition, these studies reported enhanced expression or activation levels of phosphotyrosine, PKC, different MAPK signaling events, and transcription factors, overall indicating the high capacity of *S. suis* to activate microglial cells. As shown with other phagocytes, CPS also contributes to *S. suis* resistance to microglia phagocytosis and regulated the inflammatory response by hiding proinflammatory components from the bacterial cell wall, while the absence of SLY did not have a major impact on *S. suis* interactions with microglia [165].

## *Streptococcus suis* interactions with dendritic cells

DCs exist in all lymphoid and nonlymphoid organs and are among the first responders to infection at the mucosal surface [211]. In steady-state conditions, mucosal DCs are continually replenished through the influx of committed DCs, as well as the local differentiation of DC precursors and immigrating monocytes. Immature DCs have a sentinel role in capturing antigens, but after encountering microorganisms, they are activated and undergo a complex maturation process characterized by the up regulation of costimulatory molecules and the production of proinflammatory mediators [211]. Mature DCs migrate to the adjacent ӏymphoid organs where they can induce antigen-specific T and B cell responses. In this respect, DCs are the most important professional antigen-presenting cells (APCs) and express 10–100 fold more major histocompatibility complex (MHC) molecules than monocytes or macrophages [212, 213]. Thus, DCs represent an essential link between the innate and adaptive immunity, and interactions between DCs and microorganisms, such as *S. suis*, strongly influence the outcome of a disease. Differently from what is observed after in vitro incubation of *S. suis* with macrophages, few bacteria are associated to mouse or pig DC surface, even after serum opsonization, and CPS might hamper the adherence of the pathogen to the cells [122, 214].

In vitro bactericidal and phagocytosis assays combined with confocal and electron microscopy have established that under nonopsonic conditions, *S. suis* serotype 2 possesses a relatively high resistance to the phagocytosis by mouse, porcine, and/or human DCs [77, 122, 214]. The internalized bacteria are destroyed in a time-dependent manner [77, 122, 214]. However, the levels of phagocytosis and intracellular survival of *S. suis* serotype 2 are higher in human monocyte-derived DCs than in porcine bone marrow-derived DCs [77, 214]. This may be due to the use of different strains and/or differences in the phagocytosis/killing capacity between the two DC types. Interestingly, *S. suis* serotype 2 can still be recovered, albeit at very low numbers, from the intracellular compartment of human DCs 24 h postincubation. As DCs traffic from the mucosa to the lymphoid tissues via bloodstream, even such reduced intracellular persistence may enable the bacteria to rapidly disseminate in the human body [77].

Mechanisms of *S. suis* serotype 2 resistance to phagocytosis/killing by DCs remain largely unexplored (Figure 1). Nonencapsulated mutant strains are readily internalized by cells independently of the presence of complete serum [122, 214, 215]. Thus, CPS may actively interfere with the phagocytic signaling machinery of those cells, similarly to what is observed during interaction of the bacteria with macrophages [168]. Indeed, beads covalently linked with purified *S. suis* serotype 2 CPS block the internalization of uncoated latex beads by mouse DCs [168]. Yet, if internalized, bacteria are easily destroyed, and neither the presence of CPS nor that of complete serum influence the rate or the kinetics of this intracellular killing [77, 214]. Whereas CPS of serotype 2 inhibits complement deposition at the bacterial surface, SLY, and cell wall modifications, including PG *N*-deacetylation and LTA *D*-alanylation, are also crucial to fully protect bacteria against complement-mediated opsonophagocytosis and killing [122]. It should be noted that the bacterial serotype as well as strain diversity within the same serotype [201, 215] might influence the interactions with DCs. While strains of serotypes 1 and 2 were relatively resistant to phagocytosis by human DCs, those of serotypes 4 and 9 were more efficiently taken up by these cells [77]. It remains to be determined whether this is related to the CPS composition or other bacterial factors inherent to each strain used [201].

*S. suis* induces production of cytokines (IL-1*β*, IL-6, TNF-*α*, IL-12p70/p40, IL-23, IL-10, IFN-*β*, G-CSF, GM-CSF) and chemokines (IL-8/CXCL8, CCL2, CCL3, CXCL1, CXCL9, CXCL10) by mouse, porcine, and/or human DCs [77, 122, 214, 216] (Figure 2). However, there are differences in cytokine production between studies, which could be due to differential activation resulting from variations in the exposition and/or the composition of bacterial surface components of *S. suis* strains/serotype used [37, 201, 215]. Similarly to what is noticed with macrophages, cell wall components may be mainly involved in this immunostimulatory activity, as nonencapsulated mutant strains globally induce higher levels of secretion of cytokines/chemokines by DCs [37, 77, 122, 201, 214]. Interestingly, cell wall modifications play a partial role in the activating capacity of *S. suis* as shown by the impaired secretion of some cytokines by mouse DCs stimulated with *ΔpgdA* and *ΔdltA* mutant strains [122, 201]. The *S. suis* capacity to activate DCs can be also affected by the CPS type. Using CPS switched mutants of a serotype 2 strain, it was shown that expression of CPS8 or CPS4 (instead of CPS2) differently modulates the induction of several cytokines [7]. Besides cell wall components, CPS promotes chemokine production by DCs, as shown by production of CCL2 and CCL3 after stimulation of mouse DCs with strains of serotypes 2, 9, and 14 [7, 37]. This finding was further confirmed when using CPSs purified from *S. suis* serotypes 1, 2, 1/2, 3, 7, 8, 9, and 14 [217, 218]. However, CPS is mainly a poor immunogenic molecule, and no other cytokine is detected in the supernatant of CPS-stimulated DCs [217, 218]. Remarkably, purified CPSs of serotypes 2 and 14 exert immunosuppressive effects on mouse DC activation, as a diminution of IFN-*γ*-induced B cell-activating factor of the tumor necrosis factor family (BAFF) expression is observed in CPS-pretreated cells [217]. This could impact on the development of the ongoing specific humoral response given that BAFF cytokine is involved in B cell proliferation, Ig class switching, and Ig secretion [219, 220]. Whereas sialic acid is suspected to contribute in immune evasion [158, 221], there is no difference in mouse DC stimulation whether the cells are incubated with purified native or desialylated *S. suis* CPSs of serotypes 2 and 14 [217].

Cytokines produced by activated DCs have a major influence on T cell polarization, differentiation, and clonal expansion. IL-12 is a proinflammatory cytokine that promotes T helper (Th) 1 cell responses, whereas IL-10 is an anti-inflammatory cytokine orienting toward Th2 or regulatory T cell responses depending on the expression of other tolerizing factors. A study comparing the interaction of different serotypes of *S. suis* with human DCs demonstrated that serotype 2 is the least effective serotype at inducing cytokine production by these cells and causes the highest IL-10/IL-12 ratio [77]. This higher propensity to down regulate the host cell-mediated response [222] can be connected to the fact that serotype 2 is the most commonly associated with invasive disease in pigs and humans. Indeed, splenic DCs derived from *S. suis*-infected mice specifically failed to secrete IL-12p70 in response to ex vivo stimulation with consequent reduction of activation of CD4+ T cells. SLY expressed by whole *S. suis* as well as serum opsonization were shown to regulate the release of IL-12p70 and IL-10 by mouse DCs [122].

TLRs have been demonstrated to be involved in the interactions between mouse DCs and *S. suis* serotypes 2, 9, and 14 (Figure 2). Firstly, *S. suis* was shown to induce the expression of TLR2 and TLR6 by porcine DCs. In addition, the release of cytokines and chemokines by *S. suis*-stimulated mouse DCs is partially TLR2 dependent, and these secretions as well as NF-κB, ERK, p38, and JNK pathways are dramatically abrogated in the absence of MyD88 [196, 201]. Recognition of lipoproteins by TLR2 induces G-CSF and IL-1*β* production by mouse DCs, indicating a major role of these bacterial surface components in APC activation [182, 194]. However, TLR4/TRIF pathway and NOD2 are only marginally engaged in DC-mediated inflammatory process [37, 182, 194, 196]. It has been suggested that recognition of the SLY by TLR4 might contribute to the induction of IFN-*β* by mouse DCs [201]. More recently, a complementary mechanism has been described whereby, following internalization and phagosome maturation, *S. suis* nucleic acids activate endosomal TLR7 and TLR9, leading to the activation of IRF1, IRF3, and IRF7 and the subsequent production of type 1 IFN [201], which in turn modulates autocrine cytokine responses. In the same study, TLR3 was also found to be partially involved in the activation of type I IFN signaling in DCs [201]. In addition, *S. suis* DNA, and, to a lesser extent, RNA, have been reported to induce G-CSF and IL-1*β* production by mouse DCs [182, 194]. Studies have addressed the role of inflammasome activation in IL-1*β* production and reported that engagement of NLRP1, NLRP3, AIM2, and NLRC4 inflammasomes was strain-dependent [193, 194]. High levels of SLY production participate in pro-IL-1*β* maturation and inflammasome activation, resulting from pore formation and ion efflux [194]. The potential contribution of other nucleic acid-sensing pathways, such as cGAS-STING, should also be considered in light of recent studies on bacterial (including streptococcal) infections and host immunity (see [223] for a review). Finally, the production of CCL3 induced by the CPS of different serotypes occurs through MyD88-dependent pathways, with a partial contribution of TLR2, whereas CCL2 production appears to be TLR independent [217, 218]. These findings suggest that other receptors, such as members of the C-type lectin receptor family, may be involved in the inflammatory response following DC interaction with CPS and/or whole *S. suis*. In vivo, during *S. suis*-induced CNS and systemic disease, neither TLR2 nor TLR4 alone was found to significantly contribute to systemic disease, supporting the involvement of other MyD88-dependent TLRs [224]. Moreover, CNS disease did not always require MyD88 signaling, indicating that additional pathways are likely involved. 

*S. suis* stimulation results in an increase in surface expression of costimulatory molecules by mouse (CD40, CD86, and MHC-II), human (CD83 and CD86), and, to a lower extent, by porcine (MHC-II and CD80/86) DCs. No specific role can be attributed to any of *S. suis* components in this activation, but CPS expression blunts human and porcine DC maturation [77, 122, 196]. Expression of surface molecules by mouse DCs in response to *S. suis* serotype 2 occurs mainly but not exclusively through a MyD88-dependent pathway. CD86 and MHC-II up regulation is partially TLR2 dependent, whereas CD40 expression is diminished in mouse DCs, which have a dual deficiency in TLR2 and TLR9. Neither TLR4 nor NOD2 is implicated in the regulation of the above-mentioned costimulatory molecules [196].

The MHC-II pathway involves a series of complex intracellular events that eventually lead to MHC-II trafficking to the cell surface. The expression of MHC-II molecules on *S. suis*-stimulated DCs has been mostly studied at long incubation times as a mere cellular maturation marker. A study provided a mechanistic understanding of MHC-II regulation during *S. suis* serotype 2 infection in vitro and in vivo. Results suggested that encapsulated *S. suis* impairs the ability of mouse DCs to increase the synthesis of new MHC-II molecules shortly after they encounter the pathogen. The fact that newly synthesized MHC-II molecules constitute the primary source for antigen presentation underscores the likelihood that *S. suis* disturbs the optimal timing between the processing of pathogen-derived antigens and MHC-II loading and trafficking [222]. Once again, serotype 2 was reported to be the least effective serotype at inducing human DC maturation in comparison with strains of serotypes 1, 7, and 9 [77]. It can be hypothesized that CPS thickness/composition may play a role in the observed differences. In addition, the role of unknown virulence factors differently expressed among serotypes cannot be ruled out.

Overall, *S. suis* uses several strategies to impair phagocytosis ability, maturation, and activation of DCs, which may result in reduced antigen processing and T and B cell activation [222, 225]. The consequences of this subversion of DC functions on the development of adaptive immune responses against *S. suis* need to be explored. Besides, *S. suis* can affect the interactions between DCs and another type of cells also involved in host defense against the bacteria, namely the NK cells. Indeed, some studies have shown that NK cells participate in the innate response to bacterial infections via the production of IFN-*γ* and TNF-*α* [226]. During *S. suis* infection, it is hypothesized that dendritic cells (DCs) first recognize the bacterium and produce cytokines, which in turn promote IFN-*γ* induction in NK cells. This process may enhance DC–NK cell interactions, potentially creating a synergistic effect on DC cytokine production [227].

## Conclusions

Significant progress has been made in recent years in understanding the complex relationships established between *S. suis* and its hosts, both animal and human. However, when it comes to the innate side of the immune response, many uncertainties still remain (Table 1 and text above). Moreover, discrepancies among studies regarding the bacterial factors involved in host cell activation and the molecular pathways triggered during *S. suis* infection likely reflect differences in experimental conditions. Variations in the host cell types used (including monocytes, macrophages, DCs, or epithelial cells) as well as their origin (human, mouse, or pig) can substantially influence the observed immune responses. In addition, the bacterial strains studied may differ in their virulence and genetic background, particularly among serotype 2 isolates, which can lead to variability in the host–pathogen interactions reported. Technical differences between studies, such as infection conditions, multiplicity of infection, and the nature of the stimuli tested, may further contribute to divergent conclusions. Importantly, in some studies the absence of appropriate endotoxin controls may lead to misinterpretation of results, as *S. suis* is a Gram-positive bacterium and therefore does not produce lipopolysaccharide; contamination with endotoxin in recombinant protein or bacterial preparations could artificially trigger TLR4-dependent responses. Finally, several bacterial factors have been evaluated primarily in their purified recombinant form, which may not fully reflect their activity in the context of intact bacteria, where expression levels, surface localization, and interactions with other bacterial components can significantly influence their biological effects. The coming years will undoubtedly bring about a wealth of discoveries, further enhancing our understanding of the intricate interactions that *S. suis* establishes with infected mammals.

**Table 1:** **Examples of gaps in our understanding of the innate immune response mounted against *Streptococcus suis***

The precise mechanisms used by *S. suis* to colonize and/or invade its hosts
Interactions of *S. suis* with the mucus of the intestinal and respiratory epithelium
Interactions of *S. suis* with the gastric environment
The influence of chemokine and cytokine secretion by epithelial cells on colonization and/or dissemination of *S. suis*
The exhaustive identification of receptors involved in *S. suis* recognition by host cell populations (e.g., epithelial cells and macrophages)
Interactions of the CPS of different serotypes and host cells
The impact of the variations in the expression and the thickness of CPS on the innate immune response
The contribution of antimicrobial peptides in the defense against *S. suis*
The complete mechanisms of *S. suis* resistance to phagocytosis by dendritic cells, macrophages, and neutrophils, including the precise role of CPS
Most of the knowledge is based on studies considering the serotype 2 of *S. suis*. Better knowledge of the interactions of other serotypes (9, 14, etc.) with the innate immune system is needed
The exact contribution of natural killer cells in *S. suis* pathogenesis
The importance of endosomal TLR7 and TLR9 detection of *S. suis* in the host defense strategy and the pathogenesis
The actual contribution of cGAS/STING pathway in the immune response against *S. suis*

*CPS* capsular polysaccharide, *TLR* toll-like receptor, *cGAS/STING* cyclic GMP-AMP synthase/stimulator of interferon genes

## Data Availability

No datasets were generated or analyzed during the current study.
